# What are the most important quality of life domains for patients with aplastic anemia and paroxysmal nocturnal hemoglobinuria?

**DOI:** 10.1007/s00277-025-06377-z

**Published:** 2025-04-29

**Authors:** Katherine J. Taylor, Susanne Singer, Saskia Langemeijer, Richard J. Kelly, Louise Arnold, Jens Panse, Christopher J. Patriquin, Jun-ichi Nishimura, Maria Piggin, Pascale O Burmester

**Affiliations:** 1 Division of Epidemiology and Health Services Research, Institute of Medical Biostatistics, Epidemiology, and Informatics (IMBEI), University Medical Centre of Johannes Gutenberg University, Mainz, Germany; 2 Department of Hematology, Radboudumc, Nijmegen, Netherlands; 3 Department of Haematology, St. James’s University Hospital, Leeds, UK; 4 Center for Integrated Oncology Aachen Bonn Cologne Düsseldorf (CIO ABCD), Aachen, Germany; 5 Department of Hematology, Oncology, Hemostaseology and Stem Cell Transplantation, University Hospital RWTH Aachen, Aachen, Germany; 6 Division of Medical Oncology & Hematology, University Health Network, University of Toronto, Toronto, Canada; 7 Department of Hematology and Oncology, Osaka University Graduate School of Medicine, Suita, Japan; 8 PNH Support UK, London, UK; 9 PNH Global Alliance, Tiel, Netherlands; 10 Stiftung Lichterzellen, Cologne, Germany

To the Editor,

Paroxysmal nocturnal hemoglobinuria (PNH) and aplastic anemia (AA) are rare, interrelated, life-threatening hematological disorders characterized by hemolytic anemia, thrombophilia, and end-organ damage alongside bone marrow failure with various degrees of pancytopenia. The incidence in Europe per year ranges from 1 to 3.5/million people [1, 2], and the prevalence is about 16/million people [3]. While new treatment options have emerged, most patients still experience reduced quality of life (QoL) [4]. However, conventional tools assessing QoL in patients with AA/PNH were designed for patients with other diseases [5]. This is problematic, as specific issues important to patients with AA/PNH are absent in such questionnaires. Other issues, typically chemotherapy-related, are likely irrelevant, potentially impacting questionnaire completion. We are addressing this gap. Extensive development work has led to the preliminary version of the QLQ-AA/PNH-54 [6, 7], an AA/PNH-specific QoL questionnaire comprising 54 items that is already in use by the scientific community [8]. We are now moving forward with the final validation stage of development.

In preparation for the validation study of the QLQ-AA/PNH-54, we created an online survey in German and English using LamaPoll (see Supplementary) [9]. The purpose was to have patients with AA/PNH select the most important QoL domains in the QLQ-AA/PNH-54. After confirming their AA/PNH diagnosis, participants were shown 12 preliminary domains from the QLQ-AA/PNH-54: physical functioning, role functioning, emotional functioning, concentration, fatigue, social support, limitations in daily activities (illness intrusiveness), managing infections, fear of progression, stigmatization, body image, and “other” problems (oral inflammation, bleeding, breathing, and sexual problems). Participants were able to view the specific QLQ-AA/PNH-54 questions underlying each domain in case they were unsure about a domain’s intent.

Participants were made aware of the survey via Stiftung Lichterzellen’s emailing list, the Facebook and Instagram accounts of the PNH Global Alliance and Stiftung Lichterzellen, and shared in three closed patient-driven Facebook groups [10]. The survey was available from 11 to 18 October 2024; one survey reminder was sent. Of 169 participants who started the survey, 146 completed it, of whom 3 indicated they did not have an AA/PNH diagnosis and were removed from the analysis. Of the 143 respondents with a self-reported AA/PNH diagnosis, the five most important domains were fatigue (71%), illness intrusiveness (68%), fear of progression (65%), physical functioning (58%), and emotional functioning (43%) (Fig. 1). Body image and stigmatization were among the top five domains for 15% and 12% of participants, respectively.

Our survey was anonymous, meaning we are unable to report demographic/clinical characteristics or make statements about subgroup differences, such as disease stage. Selection bias is possible due to the survey’s online format. It is also possible not all participants were patients with AA/PNH; however, the survey targeted groups dedicated to this patient population, and it is unlikely that individuals unconnected to this rare disease would have been motivated to complete it. Our survey highlights QoL domains prioritized by patients with AA/PNH, which could contribute to the continuing education of patients and families about expectations and effects of treatments, in particular regarding fatigue as a dominant concern.

**Fig. 1:**
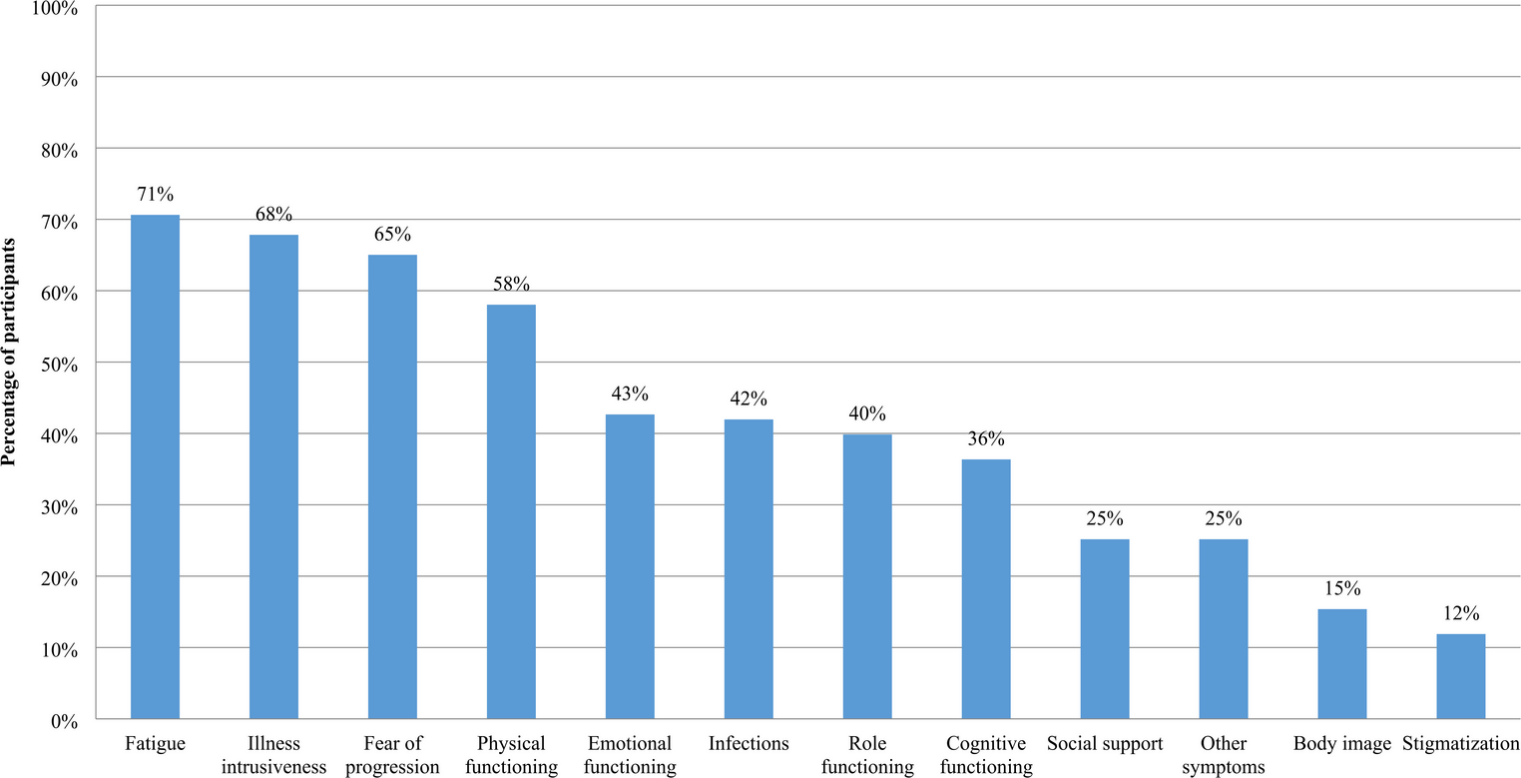
Survey respondents’ selection of their top five most important issues related to aplastic anaemia and/or paroxysmal nocturnal hemoglobinuria (*n* = 143)

## Electronic supplementary material

Below is the link to the electronic supplementary material.

Supplementary Material 1

## Data Availability

The data reported here are available from the corresponding author.
