# Supplementary material for: What are the most important quality of life domains for patients with aplastic anemia and paroxysmal nocturnal hemoglobinuria?
Source: Ann Hematol. 2025 Apr 29;104(5):3073–5. doi: 10.1007/s00277-025-06377-z (PMC12141162; doi:10.1007/s00277-025-06377-z)
Supplement: Supplementary file 1 — Supplementary Material 1 [file 277_2025_6377_MOESM1_ESM.pdf]

The following is a copy of the English survey. Any text in italics has been added to provide explanations to the current reader that are no longer obvious now that the survey is offline.

[Page 1 of the survey]

As part of the final phase in the development of a questionnaire to assess the quality of life of patients specifically with aplastic anaemia (AA) and/or paroxysmal nocturnal hemoglobinuria (PNH), we would like to show you a list of is- sues faced by AA/PNH patients and ask your opinion on which ones are the five most important issues for you.

Your reply will help us design our plan for the final phase of the questionnaire development. This survey is solely for patients with a diagnosis of AA and/or PNH.

Have you been diagnosed with Aplastic Anemia and/or Paroxysmal Nocturnal Haemoglobinuria?*

- ◯ Yes
- ◯ No

[Only participants who indicated "yes" to the question above moved on to the second page of the survey.]

[Page 2 of the survey]

We would like to ask you which five of the following issues are most important for you. We understand that more than five of the issues are likely to be important to you, **but please select the five issues that are most important for you in relation to your diagnosis of AA or PNH**.

If you scroll over the information icon, you will find questions to help you understand what is meant by each issue.

Which five issues are most important for you in relation to your diagnosis of AA or PNH?

- [ ] Physical limitations ℹ
- [ ] Fulfilling your roles in life ℹ
- [ ] Coping emotionally ℹ
- [ ] Concentration ℹ
- [ ] Fatigue ℹ
- [ ] Support by friends, family and through interaction with other patients ℹ
- [ ] Limitations to your activities and daily life due to the illness ℹ
- [ ] Managing infections ℹ
- [ ] Worries and fears about your illness becoming worse ℹ
- [ ] Feeling stigmatized due to your illness ℹ
- [ ] Feelings about your appearance ℹ
- [ ] Other problems that can occur ℹ

Please click "Next" to ensure your answers are save

[See the additional information at the end to see what the patients could see

under each “i” symbol]
[Page 3 of the survey]

Thank you for your participation!

By participating in this survey, you are helping IPIG (International PNH Interest Group) validate the AA/PNH QLQ-54 questionnaire, which is a disease-specific quality of life questionnaire designed to comprehensively assess the quality of life of people suffering from AA and PNH.

If you have any questions please contact us at contact@pnhga.org.

PNH Global Alliance
&
IPIG

Survey created with
LamaPoll

[The following two pages show what details that patients could see by hovering over each "i" symbol on page 2 of the survey. This way, the patient could gain a better idea of that was meant by the short issue description.]

Issue	Questions that were visible under the "i" for the specific issue.
Physical limitations	Has going for a long walk caused you difficulties?
	Have you had difficulty climbing stairs?
	Have you had difficulty standing for an extended period?
Fulfilling your roles in your life	Have you still been able to go on holiday as you wished?
	Have you had problems coping with the household chores?
	Have you had no energy left for your personal life and hobbies?
	Have your work or other daily activities been restricted?
	Have you been able to do what you wanted?
	Have you felt that you were missing out on something in life?
	Have you been proud of what you achieved despite the illness?
Coping emotionally	Have you been irritable?
	Have you felt vulnerable?
	Have you felt at the mercy of your illness?
	Have you worried a lot?
	Have you felt depressed?
Concentration	Have you had difficulty concentrating?
Fatigue	Have you felt tired?
	Have you had to rest?
	Have you been exhausted for days after you exerted yourself?
	Have you had difficulty getting out of bed in the morning?
	Have you been unable to bring yourself to do things or have you been apathetic?
Supported by friends, family and through interaction with other patients	Have you felt supported by friends and family?
	Have you missed the interaction with other patients?

Issue	Questions that were visible under the “i” for the specific issue.
Limitations to your activities and daily life due to the illness	Has it bothered you that you had to look out for minor symptoms because they could mean something bad?
	Has your everyday life been affected by pain?
	Has it been a problem for you to ration your strength?
	Have you found it a problem to give up sporting activities?
	Has it bothered you that you were unable to make plans?
	Has it bothered you that you had to be careful?
	Has your normal routine been disrupted?
	Has it bothered you that you were unable to be spontaneous?
	Have you had signs (e.g. pallor, bruises, dark urine, yellow skin) that repeatedly reminded you of your illness?
Managing infections	Have you had problems with susceptibility to infections?
	Have you had to take care all the time to avoid picking up infections?
Worries and fears about your illness becoming worse	Have you been concerned that there might not be any more therapy for you?
	Have you been troubled by thoughts of an uncertain future?
	Has everything revolved around your illness?
	Have you been afraid of a deterioration in your blood count?
	Have you been bothered by your blood count results?
	Have you been afraid that therapies might not work?
	Have you been afraid of a relapse or deterioration?
Feeling stigmatized due to your illness	Has it bothered you that your relatives were upset by your illness?
	Has it annoyed you that you had to explain yourself, e.g. why you have been unable to do this or that?
	Has it bothered you repeatedly having to face up to your illness?
	Has it bothered you to be classified as ill?
Feelings about your appearance	Has the illness made you feel less attractive?
	Have you felt good about your body?
Other problems that can occur	Have you been less interested in sex?
	Have you been less able to enjoy sex?
	Have you had problems with swelling or inflammation in the mouth?
	Have you had problems sleeping?
	Has your body felt heavy?
	Have you been short of breath?
	Have you had a tendency to bleed?
